# Using a 3 stage process to create a consumer research contact list in a paediatric health setting: the PARTICIPATE project

**DOI:** 10.1186/s40900-021-00300-2

**Published:** 2021-08-07

**Authors:** Fenella J. Gill, Catherine Pienaar, Tanya Jones

**Affiliations:** 1 Perth Children’s Hospital, Child and Adolescent Health Service, Hospital Avenue, Nedlands, WA 6009 Australia; 2 School of Nursing, Faculty of Health Sciences, Curtin University, Perth, Western Australia Australia; 3 Curtin enAble Institute, Faculty of Health Sciences, Curtin University, Perth, Western Australia Australia; 4 School of Allied Heath, Faculty of Health Sciences, Curtin University, Perth, Western Australia Australia; 5 Formerly of the Consumer and Community Health Research Network (Now named Consumer and Community Involvement Program), Harry Perkins Institute of Medical Research, Level 6, 6 Verdun Street, Nedlands, WA 6009 Australia

**Keywords:** Paediatric, Child health research, parents, research participation, Stakeholder, Consumer and community involvement

## Abstract

**Abstract:**

The impact of child health research can be far reaching; affecting children’s immediate health, their adult health, the health of future generations and the economic wellbeing of countries. Consumer and community involvement is increasingly recognised as key to successful research recruitment. Systematic approaches to research recruitment include research registries or research contact lists.

**Objective:**

Develop a process of creating a consumer research contact list for participating in future research opportunities at a children’s health service.

**Methods:**

A healthcare improvement approach using a 3 stage framework; 1) evidence review and consultation 2) co-production of a research communications plan with stakeholders (including consumers), including a draft research information brochure 3) prototyping involved iteratively testing the brochure, surveying parents or carers who attended outpatient clinics or the hospital Emergency Department, and conducting follow up telephone calls.

**Results:**

There was overall support for the creation of a research contact list, but some unknowns remain. 367 parents or carers completed the survey and 36 participated in a follow up telephone call. Over half would be willing to join a research contact list and more than 90% of the children of parents or carers surveyed were not currently participating in research. Several potential barriers identified by health service staff were dispelled. Research communications and a future contact list should be available in electronic form.

**Conclusions:**

There was strong support for creating a research contact list. The approach will inform our future directions including creation of an electronic research contact list easily accessible by consumers of the children’s health service.

**Plain English Summary:**

Recruiting enough children to participate in research studies can be challenging. Establishing a registry or list of young people willing to be contacted to participate in research is one way of addressing this problem. At our children’s health service, we wanted to explore the idea of developing a research contact list and we were particularly keen to involve consumers and community members in this process, which involved: 1.Reviewing other examples of research contact lists and consulting with a range of people, including consumers and community members, 2. Co-producing a research communications plan with parents, young people, health service staff and research staff, including a draft research information brochure for families, and 3. Testing the acceptability of the brochure by surveying parents or carers who attended outpatient clinics or the hospital Emergency Department, and conducting follow up telephone calls with them. 367 parents or carers completed a survey and 36 participated in a follow up telephone call. Over half were willing to join a research contact list and more than 90% of the children of parents or carers surveyed were not currently participating in research. Several potential barriers raised by consumers and health professionals in the first stage of the project were not found to be a concern for the parents or carers surveyed. Responses showed research communications and a future contact list should be available in electronic form**.** These findings will inform the future creation of an electronic research contact list, easily accessible by consumers of the children’s health service.

## Introduction

The impact of child health research can be far-reaching; affecting children’s immediate health, their adult health, the health of future generations and the economic wellbeing of countries [1]. Despite this, children are underrepresented in clinical research [2]. Opportunities to involve children in research today are much greater [1], yet recruiting paediatric research participants remains challenging. Specific challenges include ethical and practical concerns about overburdening parents in acute and emergency healthcare contexts where families may be experiencing intense emotional or psychological stress [3]. Recruiting insufficient research participants can be a barrier to conducting research and result in waste of research resources [4–6].

Research registries are an emerging strategy to optimise participant recruitment [5, 7]. A registry is an organised system to collect uniform data to evaluate specified outcomes for a population [8]. Clinical or hospital-based registries, are used to collect patient information, improve quality of care [9] and to study specific conditions [10]. Population-based patient registries can be interlinked with other databases and include entire nations. For example, although not primarily designed to enable access to research participants [5], the Danish National Patient Registry has been used to collect administrative and clinical data from all hospitals since 1978 [11]. Data are used extensively for research and can be linked at the patient level with other registries, other trials, and population surveys. In Australia, national population-based registries also exist [12, 13]. Unlike a registry, a research contact list comprises just contact information and permission to approach to take part in research.

### Consumer and community involvement in Health Research

The involvement of ‘consumers and the community’ or ‘patients and the public’ is increasingly recognised as important in healthcare decision making [14], as well as design and conduct of research [15–17] and setting of research priorities [18]. Consumer and community involvement (CCI) ensures that research focuses on issues relevant to the public [19] and has been shown to have an impact at all stages of research [20]. However, it needs to be genuine and more than a box-ticking exercise. Whilst patient engagement has become integrated into organisations and is often a pre-requisite for funding, tokenism or superficial involvement can occur when consumer input is not reflected in decisions made about the research [21].

In Australia there is also recognition of the importance of CCI in determining what is researched and how it is undertaken [22], and the National Health and Medical Research Council provides guidance on [23] and promotes active involvement of consumers and community members in health and medical research. In the Western Australian (WA) context, CCI in research is facilitated by the Western Australian Health Translation Network (WAHTN) [24] through the Consumer and Community Involvement (CCI) Program (formerly the Consumer and Community Health Research Network) by researchers partnering with consumers and community members to make decisions about health research priorities, policy and practice. The WAHTN aim is to embed CCI as standard practice in local health research [25]. The CCI Program explored CCI from the perspectives of WA researchers, reporting that consumers provide unique perspectives on research and can identify issues that may not be obvious to researchers. They also highlighted that consumer endorsement of research is vital to achieve community support [26].

### The PARTICIPATE project

At the West Australian Child and Adolescent Health Service, there was no mechanism for families to express their wishes to be contacted about research opportunities. There was a need to address this gap and improve the health service’s research capacity in a manner that was feasible and acceptable to all stakeholders including staff as well as consumers and community. The initial purpose of the PARTICIPATE project was to explore the possibility of development of a research registry.

## Methods

**Fig. 1:**
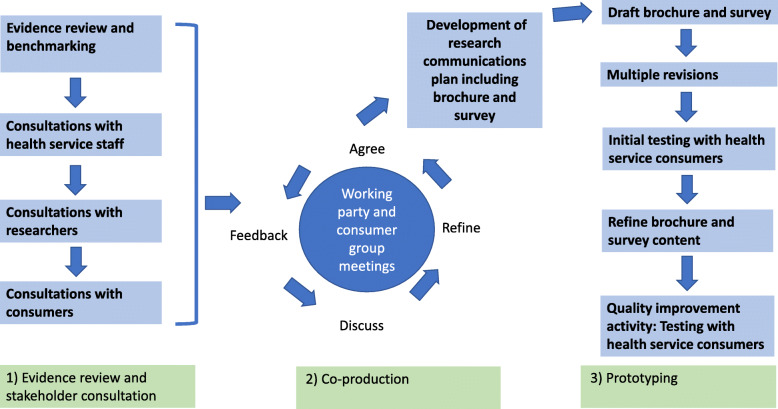
A three-stage framework, adapted from Hawkins et al. [27], consisting of 1) evidence review, benchmarking and stakeholder consultation, 2) co-production and 3) prototyping

The healthcare improvement project approach involved using a three-stage framework, adapted from Hawkins et al. [27], consisting of 1) evidence review, benchmarking and stakeholder consultation, 2) co-production and 3) prototyping (See Fig. 1). The methods used at each stage facilitated the iterative process of integration of evidence and stakeholders’ knowledge, expertise and preferences. The headings used throughout follow the flow chart steps in Fig. 1. In this project we have used the term ‘co-production’ to describe our process of working in equal partnership to plan and undertake research with people who have lived experience of the health service ie the health service staff as well as consumers and community members. Health service governance approval was obtained for Stage 3 of the project only, as the purpose of consultation undertaken in the first two stages of the project was to actively involve consumers, community members and health service stakeholders in the planning and development of the research, which does not require Ethics Committee approval [28, 29] The SQUIRE 2.0 (Standards for Quality Improvement Reporting Excellence) publication guidelines were followed [30].

### Stage 1: evidence review, benchmarking and stakeholder consultation

The overall aim of Stage 1 was to both learn from what had been previously reported and to gather the perspectives of multiple stakeholders, including consumers, about the issues related to establishing a research registry in our context.

#### Evidence review

An iterative literature search was conducted prior to the consultations taking place. At this early stage we envisaged establishing a research registry would be a straightforward process, and the focus was on the logistics of registries in terms of databases, consenting processes and lessons to learn. A focused search was conducted to address the following two questions; In healthcare settings, what initiatives have been used for research participant recruitment? and; In healthcare settings, how have research registries been implemented and evaluated? Proquest and PubMED databases were searched. Key terms were as follows: Registr*, System*, Opt-in, Research consent system, Patient* consent, clinical registr* and paed*. Included were full text, peer-reviewed publications in English between 2013 and 2018. Additional strategies included searching end-text reference lists of identified articles and searching of key websites. Results of the search identified 256 articles from Canada, United States (US), Europe, United Kingdom (UK) and Australia. Articles included descriptive reports, qualitative and quantitative studies, and systematic reviews. For benchmarking, we explored Australian paediatric hospitals’ websites for research registry personnel contact details and attempted to contact individuals.

#### Consultation

Consultations were held with stakeholders who included consumers, health service staff and other researchers. We adopted a practical approach to facilitate consultation and ensure the consumer voice was heard throughout. A project steering group of 25 key stakeholders consisted of health professionals, researchers, information technology professionals, legal services professionals, the health service communications team, health service clerical staff and a consumer engagement team member. Table 1 further details consultation participants.

**Table 1:** Steering group, working party, Nursing Research Consumer Advisory Panels and consultation participants

Groups	n
**Steering group**	25
Health professionals; Hospital, Community Health, Mental Health	
Clinical researchers	
Patient management system professionals	
Health information management system professionals	
Health service business intelligence professional	
Consumer Engagement team member	
**Working party**	6
Researchers	
Health professionals; Hospital, Community Health, Mental Health	
Working with	
Nursing Research Consumer Advisory Panel	6
Nursing Research Youth Advisory Panel	7
Communications team	1
**Consultations**	
Health professionals; Hospital, Community and Mental Health	57
Health Service Research and Research Governance professionals	12
Patient management systems professionals	35
Health Information management system professionals	3
Information and Communication Technology professionals	2
Legal services team	2
Communications team	5
Business Intelligence professional	1
Consumer and Family Engagement team	3
Community Conversation participants	26
Research Youth Advisory Panel	7
Research Consumer Advisory Panel	6
Health Service Consumer Advisory Council	6
Professionals from other organisations	13

Initially a targeted approach was used to identify health service groups and individuals who had an interest in, and who might affect or be impacted by establishment of a research registry. In collaboration with the steering group, we created an initial list of people and departments who would be impacted by or would likely wish to provide input about creating a research registry. We contacted them by email or telephone arranging to meet in person or consult by telephone. As further people were identified and recommended by others we expanded the number and range of consultations. The aim was to gather multiple perspectives about possible solutions and to identify barriers to develop a feasible process to create a registry. These unstructured consultations were undertaken as individual interviews or small group meetings where the topic of creating a research registry was broadly introduced and individuals had the opportunity to provide their input. For some consultations eg. With information technology professionals, questions were more targeted to understand the capacity to integrate a research registry into the health service’s existing systems. The consultations were verbal with field notes taken. A written record of key points from each consultation was returned to people to confirm. Some people provided written input by email. The key points were collated to identify similarities and differences across perspectives and to inform subsequent consultations.

We consulted researchers at local private and public hospitals, including research directors who shared their insights, experiences, views and ideas. We found one private hospital had an existing research registry using an opt-out process, administered by clerical staff during admission of patients, however, the completion of the consent field in the database was not a mandatory field and consequently not consistently captured. We also sought input from researchers at one public hospital who had previously held a consultation with consumers and community members to explore their perspectives on an opt-out approach to using routinely collected health information for low risk research [31].

In addition to the consumer engagement team member in the steering group, our CCI strategy included regular engagement with our Nursing Research Consumer Advisory Panels (consisting of parents and young people who had utilised the health service) and the health service Consumer Advisory Committee. We also held a “Community Conversation”; a consumer and community event involving young people and parent users of the health service.

We worked together with the CCI Program team [25] to host the ‘Community Conversation’ using a World Café format [32]. The Consumer Advisory Panels advised on the planning and promotion of the event, and the wording of the discussion questions. Attendees were accessed through social media platforms and the CCI Program consumer database. Seated in small groups in a welcoming “café”-style environment, 16 parents/carers and 10 young people discussed the following three questions: what are your thoughts about a research registry?; how and when would you prefer to be approached to join a registry, and who should approach you to join the registry?; and what information should be collected and how should it be managed? To avoid impeding the flow of conversation, consumers did not move tables in between questions, as is the standard process for the World Café methodology, and most of the young people were seated at one table. After the three rounds of questions, a spokesperson from each table shared insights and summarised the conversations. Facilitators and note-takers were situated at each of the tables and later compared notes, synthesised and agreed on the key findings.

### Stage 2: co-production

Following the consultations we reflected on the issues raised and how we had not anticipated the extent to which the consultations would shift the onward direction of the project. Indeed, the findings from our preliminary review of literature had not revealed the issues which were highlighted as important to consumers and community members. The next step was to address the consultation issues raised of the need to better communicate research information to health service consumers.

In Stage 2 Co-production, a smaller working party, (who reported to the Steering group), was established consisting of the research team and health professionals representing the three areas of the health service (hospital, community health and mental health). The research team also met separately with the health service communications team and the Consumer Advisory Panels. Collectively all of these groups guided the development of a research communications plan which included three key strategies, namely, a social media campaign, messaging throughout the health service and developing a prototype research information brochure.

The co-production process involved a series of meetings to develop an information brochure, titled ‘What is child health research?’, and a survey. There were iterative rounds of discussion, rewording and redrafting using multiple mediums (in-person meetings, email communication, and voting) until agreement was reached. Consideration was given to balancing of clear messaging to a mixed audience of varying levels of health literacy whilst providing sufficient detail in the brochure to achieve the health service duty of care regarding full disclosure of information. The brochure contained information to address issues raised at the Community Conversation such as an explanation of child health research, the purpose and planned process of creating a research contact list, the information to be collected and how patient privacy would be managed. It was agreed that the term ‘research contact list’ be used to reflect the collection of consumer contact details only at this point (not linked to health information). Initial survey items were created and further developed in Stage 3. Brochure content and survey item refinements were made, presented and discussed amongst the working party and separately with the Consumer Advisory Panels and the Communications Team until agreement was reached about the content and presentation and the wording was considered clear. This stage resulted in the development of a prototype colour brochure [33] and a draft survey presented in English language.

### Stage 3: prototyping

Using the survey developed in Stage 2, the aim of Stage 3 prototyping was to assess health service consumers’ views about:

- The suitability of the draft brochure
- Being contacted about research and being part of a possible future research contact list
- A number of uncertainties regarding potential consumer participation in the research contact list; including understanding consumer willingness to join a research contact list

Objectives were to:

- Assess appropriateness of approaching families to discuss research in the hospital settings of outpatient clinics and the Emergency Department
- Assess content, clarity and presentation of the prototype brochure using a survey
- Measure families’ recall and satisfaction with their responses and obtain feedback and suggestions to refine the brochure.

Informed by the Consumer Advisory Panels, the working party guided Stage 3 prototyping which was conducted as a quality improvement activity. It was agreed the planned activity would involve requesting consumers to complete an online survey and agree to receiving a telephone call. Participation was voluntary, and the survey did not collect any identifying information unless participants agreed to be contacted for telephone follow-up and provided their name and telephone number. Health service governance approval was obtained (Reg No 29335).

#### Setting

The quality improvement activity was conducted at a 250 bed specialist children’s hospital serving a population of 500,000 children and young people. During 2018/19 there were 227,337 outpatient clinic attendances and 67,592 Emergency Department attendances [34]. Participants were recruited while attending a wide range of outpatient clinics including medical, surgical, and dental clinics. Emergency Department participants were recruited from areas in the waiting room catering for patients who had been triaged as needing less urgent care. Participants who could speak, read and understand English were included. Data collectors were experienced in communicating with families in healthcare settings. Parents or carers with acutely ill children or who appeared to be anxious or distressed were not approached. Data collection was coordinated with clinical and clerical staff to minimise disruption to workflow.

#### Planning

The 20 item survey was initially tested with 40 families who attended the children’s hospital and a community health clinic during an annual week-long patient engagement event. The testing resulted in minor wording changes to improve item clarity. The iterative testing process resulted in an additional question to understand families’ preferences about being contacted about research by health service researchers only, or by researchers from other organisations. This was to answer another uncertainty about whether families’ preference may differ depending on the research affiliation. The first 11 survey items captured participant characteristics; see Table 2 for details There were six items addressing key issues raised during consultations including willingness of families to be part of a contact list for future research studies, preferences for how to be contacted, preferred frequency of contact, the preferred person to inform families about the contact list and feasibility of the proposed process. The final four items requested participants to rate (using a 5-point Likert type scale from strongly agree to strongly disagree) their level of agreement about the brochure and the online survey content and presentation.

**Table 2:** Survey responses

Survey items	Responses n (%)
Adult age (years)	(*n*=367)
<18	6 (1.63)
19-25	14 (3.81)
26-35	116 (31.61)
35-49	202 (55)
50 or older	29 (7.9)
Identify as Aboriginal or Torres Strait Islander	
Adult	10 (2.7)
Child	22 (6.1)
Language spoken at home	
English	329 (89.7)
Other: Mandarin/Cantonese (*n*=6) Arabic (*n*=4), Mayalam (*n*=4), Punjabi (*n*=3), Nepalese, Shona, Somali, Spanish and Tagalog (*n*=2 each), and Bengali, Gujarati, Japanese, Khmer, Kiswahili, Luganda, Maori, Portuguese, Sinhala and Tamil (*n*=1 each)	38 (10.3)
Child age (years)	n (=361)
<1	41 (11.4)
1-5	113 (31.3)
6-9	72 (19.9)
10-13	93 (25.8)
14 or older	42 (11.6)
Child is currently participating in research	
Yes: oncology, allergy and immunology, respiratory, longitudinal cohort, epilepsy, renal, genetics	25(6.9%)
Please indicate your willingness to be part of a future research contact list	(*n*= 359)
Yes	196 (54.6)
Undecided	57 (15.9)
I need more information before I decide	27 (7.5)
No	79 (22)
You will only be contacted about research approved by the child and adolescent health service research ethics committee. Please indicate which organisations’ research you would be happy to be contacted about:	
The Health Service	183 (97.3)
The Health Service or the co-located Research Institute	171 (91.9)
Any collaborating organisation	154 (82.4)
Preferred information delivery methods about future research studies (more than one response was allowed)	(*n*=342)
^a^Email	212^a^
^a^Social Media (PCH Facebook=57 Instagram= 40 YouTube =14)	111^a^
^a^Text message or phone call	78^a^
^a^Information screens in hospitals	51^a^
^a^Self check-in kiosks in hospitals	27^a^
Brochures (not specified as paper or electronic)	130
Letters	91
Outpatient visits	63
During hospital admission or discharge	54
Advertising e.g. TV, billboards, buses	25
Banners in the hospital	24
Newspapers	15
Maximum number of studies to be contacted about in 1 year period	(*n*=353)
1	86 (24.4)
2	71 (20.1)
3	32 (9.1)
4	4 (1.1)
5	4 (1.1)
>5	21 (6)
Unsure	83 (23.5)
Does not want to be contacted	52 (14.7)
Preferred person with whom to have research conversation (more than one response allowed)	(*n*=352)
Any of the listed Health Service staff	164
Doctor	112
Researcher	67
Nurse	66
Allied health professional	42
Someone I have seen before at the clinic	25
Administrative staff such as clinic receptionist	13
I do not want to be asked	43
Other specified - Unsure	1

^a^Total for all electronic methods of communication

The telephone follow up consisted of four closed questions capturing participants’ recall about receiving the brochure, whether they would have liked additional information, whether they remained satisfied with the response given to being contacted about research, and their knowledge of how to update a change of preference about being contacted for research. The final question was open ended and invited suggestions to improve the delivery of information in future.

#### Testing

One of two data collectors approached families as they waited for their outpatient clinic appointment or in the Emergency Department triage waiting room. Participants who agreed to complete the survey were invited to participate in a follow up phone call. These two settings were selected to access a large volume of families who may not have previously been exposed to research and to assess the appropriateness of approaching families in these settings.

#### Evaluation

Data were collected in 2 parts between July and August 2019.

Part 1. Each participant was provided the brochure to read and invited to complete an online survey. The brochure was available to be taken away and some families did take a brochure. Survey responses were entered by participants on a tablet using Survey Monkey© software. There was no time limit to complete the survey.

Part 2. Two to 3 weeks later a telephone call was made to evaluate the experience of participants in Part 1. A maximum of three attempts to contact participants were made over a seven-day period. The purpose of the phone call was to capture participant recall of receiving the brochure, completing the survey, and to obtain feedback to improve the brochure.

### Analysis

For the survey and telephone call responses, descriptive data analyses were undertaken using frequencies and examined for distribution and percentages. Categorical data are presented using proportions and frequencies.

## Results

### Evidence review and stakeholder consultation

#### Review and benchmarking

In the first step of stage 1: evidence review and stakeholder consultation, the review findings confirmed there are many challenges to recruiting research participants, some unique to acute pediatric healthcare settings [3, 35]. Difficulty recruiting participants is a common reason for discontinuation of trials. For example, of 559 paediatric randomised control trials, recruitment difficulties were cited in 37% of the 104 discontinued trials [35]. Further, of 3428 United States (US) closed studies, 152 were terminated before completion, with 83 of these reporting termination was due to insufficient recruitment [36]. A UK review of child health research found less than 5% of registered studies involved children, and less than 2.5% of 2 million National Health Service paediatric patients were recruited into research studies [1]. A plethora of strategies have been used to improve research recruitment. A systematic review identified 72 strategies, with three that demonstrated good levels of evidence [37]. Only two strategies effectively improved recruitment: conducting open trials rather than blinded placebo-controlled trials and following up postal invitations with telephone reminders [37]. In an attempt to further understand recruitment challenges, interviews with researchers identified four factors that were positive influences; an infrastructure supporting researchers’ access to potential participants, study design, if the treating doctor mentioned the study to potential participants, and participants being motivated by altruism [4]. Recruiting research participants is complex, even more so in the paediatric setting, and successful recruitment requires a systematic approach to be followed.

A consumer research register is one systematic approach to use [5, 35]. The feasibility of an Australian ‘consumer registry’ to facilitate direct patient recruitment from hospital populations was assessed in New South Wales hospitals [5]. A survey measured consent rates, preferred methods and frequency of contact, and the feasibility of establishing the register. The concept of a register was found to be feasible, with most participants willing to be contacted multiple times utilising methods such as email [5].

Benchmarking yielded limited additional information. At the Children’s Hospital in Victoria, a component of the Electronic Medical Record includes a patient portal for families to register interest for research contact [38]. We were unable to verify how well utilised this capability is, or to find information about other paediatric hospital research registries. We did not identify any existing registries to model for our context.

Five hundred and fifty five parents or carers were approached and 367 (70.3%) agreed to participate, read the brochure and completed the survey. Three hundred and twenty six (88.8%) participants had attended outpatient clinics and 41 (11.2%) had attended the Emergency Department. In the Emergency Department there were several parents or carers who appeared anxious and the data collectors did not approach them, for example, potential participants who did not make eye contact or appeared to turn away. One participant stated that he did not feel the Emergency Department setting was conducive to being asked to read a brochure and complete an online survey. No parents in the outpatient clinic setting appeared overtly anxious.

#### Consultation

In total, more than 80 meetings were held with individuals and groups, of which a quarter were representative of the patient and carer population. We found strong consumer and health service support for establishing a systematic process to contact families about research and learnt that families are generally motivated to participate in research by altruism, and a desire to improve future health outcomes. We found that families would prefer to manage their own registry preferences using online technology. Importantly, we learned we had incorrectly assumed that consumers already understood research and what research participation involved. In addition, consumers identified that the term ‘registry’ was unclear and that this may be a barrier for some. Following the Community Conversation event, we sought advice from the Consumer Advisory Panel members who recommended using the term ‘contact list’ instead. They confirmed t there was a need to better communicate with consumers and the community about the research conducted by the health service and about research participation. Barriers to creating a research registry had mostly been raised by health service staff who expressed views that consumers would be too concerned about their privacy to wish to join a contact list, would be overburdened, were unlikely to join a contact list if their own doctor did not suggest it, or would be concerned about how to update changes. Other health service staff concerns were about using existing service-based information technology systems that were not designed to be used for another purpose, uncertainty about governance and legal requirements for patient privacy, using families’ contact details for secondary purposes other than clinical care, and the need for a research registry to be sustainable (not dependent on specific project funding or individuals).

The Community Conversation identified seven key issues relevant to establishing a research registry. These were; foreseen concerns, benefits, consent methods, raising awareness, approaching participants, information collection and information management. Logistical issues foreseen by consumers included how participant updates would be managed, such as ‘unsubscribing’ if a person no longer wanted to be part of the registry, managing data once a child turns 18 years, and knowing who would have access to their data. Registry benefits included facilitating research to increase knowledge to benefit others in the future, despite no direct benefits for participants. Most consumers supported an opt-in approach to consent. Importantly the group highlighted that adequate information was key to raise consumer awareness about research and a research registry at the health service. Consumer input regarding how best and, who would be the most appropriate person to approach families to join the registry, revealed a range of potentially acceptable options requiring further consideration. Lastly, consumers emphasised the need for consistent transparency about the collection and management of personal information. Although there was broad agreement supporting the use of health information for research, consumers felt that the general public may be reluctant to adopt an opt-out approach, unless this was preceded by a concerted consumer engagement and public awareness campaign.

### Prototyping

#### Part 1 survey

Most participants (342, 93.4%) were parents of a child attending the clinic or Emergency Department. The majority were female (299, 81.5%) and in the 36–49 years age group (202, 55%). Ten (2.7%) parents or carers and 22 children (6.1%) identified as Aboriginal or Torres Strait Islander. Most (287, 79.1%) were also primary carers of other children under 16 years. English was the primary language spoken at home for most (329, 89.7%) families. The remaining 38 (10.3%) identified 20 other languages as the primary language at home. Most frequently reported were Arabic, Mandarin and Mayalam. There were 25 (6.9%) children currently enrolled in research studies.

Four items received responses from all participants. Item response rates decreased progressively, with the final five items skipped by up to 25 participants. The item most commonly skipped (25, 6.8%) was the question ‘what is the best way we can provide you with information about being contacted for future research?’ Survey responses are presented in Table 2.

The key survey item assessed participant willingness to be part of a future research contact list. More than half (196, 54.6%) were willing to join a contact list, 57 (15.9%) were undecided, 27 (7.5%) wanted more information, 79 (22%) indicated they did not want to be part of a contact list and eight skipped this question. For those willing to be part of a future research contact list, most (154, 82.4%) did not have a preference whether researchers from the Health Service, the co-located research institute, or any of the Health Service’s collaborating partners contacted them about future research studies. Participants preferred to receive information about research electronically. Email information delivery was most popular (212, 62%). Eighty six (24%) participants indicated a preference for a maximum of one annual contact, 71 (20%) selected two, and 61(17%) were willing to be contacted more than three times in a one-year period. There were 83 (24%) participants who were uncertain. Almost half of the participants (164, 47%) did not indicate a preference for a specific health service professional to contact them about research, and approximately one third (112, 32%) preferred contact to be by a doctor.

Responses to the acceptability items indicated that the brochure and survey were both appropriate for the purpose with the right amount of information and content. Table 3 shows the responses.

**Table 3:** Feasibility and acceptability of brochure and survey

Feasibility and Acceptability	Rating scale n (%)				
Statement	Strongly agree	Agree	Unsure	Disagree	Strongly disagree
Information in brochure was easy to understand	151 (43.8)	185 (53.6)	5 (1.5)	1 (0.3)	3 (0.9)
Amount of information in brochure was just right	116 (33.7)	215 (62.5)	8 (2.3)	3 (0.9)	2 (0.6)
Survey questions were easy to answer	177 (51.3)	161 (46.7)	5 (1.5)	1 (0.3)	1 (0.3)
I am glad I took part in the survey	117 (34.0)	200 (58.1)	24 (7.0)	2 (0.6)	1 (0.3)

#### Part 2 telephone follow up

Telephone calls were placed a mean of 19 days following completion of the Part 1 Survey. Thirty six parents provided telephone feedback. Over 80% (29) were successfully contacted at the first attempt. All remembered receiving the brochure. Almost all (35, 97%) were satisfied with their response to the survey question about being part of a research contact list. Almost all (35, 97%) said that there was no other information they would have liked to receive at the time. Twenty spontaneously provided positive feedback about the brochure, indicating that it was informative, comprehensive and easy to understand. Seventeen (47%) reported they knew how to contact the health service to update contact list preferences and 19 (53%) said they did not know. Of those who did not know, six (31%) indicated they would contact the hospital. A few parents suggested shortening and refining the brochure. They indicated that more concise information would be more appropriate for families with lower health literacy. It was also suggested that we should provide links for electronic access to information.

## Discussion

We adapted a three-stage framework [27] approach consisting of 1) evidence review and stakeholder consultation, 2) co-production and 3) prototyping to test a process of creating a contact list for future research opportunities at a children’s health service. The preliminary evidence review was of some value in terms of providing background information. The stakeholder consultation stage was key in not only understanding the potential level of support for establishing a paediatric research contact list, but also identifying a considerable number of unanticipated issues for consideration and potential barriers. This led to revising our original aim and an iterative co-production prototyping process of developing and testing a research information brochure. The survey and follow up telephone call findings enabled us to answer a number of unknowns and address some potential barriers.

Most barriers were presented by the health service staff who expressed their own views about consumers’ preferences. A number of these issues had been previously reported [39] and reflected the need for an organisational cultural shift to be open to change and responsive to consumer demand. It was discovered that before establishing any registry there was a need to better inform health service consumers about research. Stage 1 consultation steered the project to include a research communications plan and informed change of terminology from research registry to research contact list.

Knowing there was a need to better communicate with consumers about research, we interpret our finding that more than 50% of survey participants indicated they would be willing to join a research contact list as relatively strong consumer support, albeit less than reported in adult hospital outpatient settings where close to 70% agreed to be contacted about future research opportunities [5]. However, an additional 23% of survey respondents reported they were undecided or wanted more information before deciding, perhaps reflecting the baseline low level of consumer and community knowledge. The prototype research information brochure was considered appropriate and useful by parents and carers. Although many parents preferred a doctor to talk to them about research participation, recruitment was perceived as a ‘team effort’ where parents did not have specific preferences about which team member talked to them about research [4]. Joining a research contact list will potentially enable greater access to research participation for families beyond the research affiliations of the treating medical team [39].

We found the outpatient clinic setting was a more appropriate environment than the Emergency Department to talk to families about research. This is likely because families experience stress and anxiety waiting in Emergency Departments and are therefore less willing to engage in dialogue with researchers [3, 40].

Our consumer population preferred research communication to be electronic rather than paper based, and for individuals to be able to manage their choices if they changed their mind about being contacted. These findings were similar to other Australian settings [5] and will inform our future directions to move to electronic systems that can be accessed by consumers. The survey showed that more than 90% of the children of survey participants were not currently participating in research and provided an indication of the potential benefit of establishing a research contact list [1].

The level of consumer and community involvement varied throughout the project. Using McKenzie and Hanley’s ladder of participation [41] participation can range from; “informed” where researchers make information available but do not seek consumer and community members’ views, “consulted” where consumer and community members views are sought with feedback offered about what has been done in response to comments, “advise” where researchers seek advice often through community forums, reference or consumer groups, “equal partners” with researchers working in partnership with consumer and community members often through membership of a steering group, with the highest level being when consumer and community members “lead the research” .

In Stage 1 Consultation we aimed for the level of “consulted” and sought the views of consumers and community members. During Stage 2 and 3 our co-production aimed for consumers and community participation as “equal partners”. In reality, the level of CCI or consumer and community participation at Stage 2 and 3 would be better described as “advise” rather than consumers being equal partners. An example illustrating this difference is that meetings were held during the day when it was not convenient for the consumers. Consumer Advisory Panel meetings were held separately after school hours or during the evening which was convenient for consumer groups but not for health professionals or other staff. For the context of this project maintaining separate groups was a practical way for all stakeholders to be involved and reflected the logistical realities of conducting research with CCI.

Limitations of the project include the focused preliminary literature review conducted to inform the work. We did not repeat or expand the search to include more recent studies as the consultations undertaken during stage 1 of the project were shaped by the findings of the literature review completed at that time. It was beyond the scope of the project to examine all concerns raised during consultation or test other aspects of the communications plan, such as the social media campaign and health service messaging. It is possible that a greater percentage of survey participants could have indicated willingness to join a research contact list if all potential concerns were addressed to their satisfaction, but including more information in the brochure may have reduced its suitability for consumers, particularly for those with low health literacy. It may also be that communication strategies beyond the information brochure could be more effective for awareness raising and addressing a range of potential community concerns [37]. However, we did seek consumer feedback on information delivery methods. We obtained responses only from participants who attended the outpatient clinics or Emergency Department. We did not assess the views of families who use the rest of the health service, which includes community health clinics and mental health services, and their views may be different. Furthermore, survey bias is likely to have impacted the responses in that those who held positive views were more likely to agree to participate.

It was also beyond the scope of the project to further examine reasons why a quarter of survey participants did not want to be part of a contact list. As previously suggested, it may be because they felt their concerns about management of their child’s health information were not adequately addressed by the brochure content, or because they were time poor. The project involved participation by English speaking participants only. The survey revealed a linguistically and culturally diverse population using the health service, with over 10% participants whose primary language at home was not English. There may have been families who declined participation because they could not speak sufficient English. Future work needs to consider this diversity and find ways to cater for the needs of all families when a research contact list is created. The survey was tested for content validity but appeared to contain too many items (the final five items were skipped more frequently than the first 16). Findings will inform future research.

## Conclusion

We used an adapted three-stage framework [27] approach to develop and test a process of creating a consumer contact list for future research opportunities at a children’s health service. Stage 1 indicated overall stakeholder and consumer support, as well as a considerable number of unknowns and potential barriers. Consumer and community input uncovered there was a need to better inform consumers about research and that terminology should be changed from research registry to research contact list. These insights steered the direction of the quality improvement activity, that is, the development and testing of a research information brochure for users of the health service, which was not initially planned. Our project illustrates both the value and practicalities of CCI from the outset of a research activity aiming to ensure the outcome is of relevance and benefit to the public.

Stages 2 and 3 enabled us to address a number of unanticipated issues and potential barriers. There was overall support to create a research contact list and most parents or carers were willing to be contacted by Health Service researchers or researchers from any collaborating organisation. Parents or carers did not have specific preferences about which research team member talked to them about research participation. Our consumer population preferred research communication to be electronic rather than paper based, and for individuals to be able to manage their participation choices. More than 90% of children of parents or carers surveyed were not currently participating in research, confirming the potential benefits of establishing a research contact list. The consumer population included people from culturally and linguistically diverse backgrounds. The quality improvement activity findings will inform our future directions towards establishing electronic systems that can be easily accessed by consumers of the children’s Health Service.

## Data Availability

Institutional governance approval does not permit sharing data.

## Abbreviations

CCI: Consumer and community involvement
US: United States
UK: United Kingdom
